# They have taken out my spinal cord: an interpretative phenomenological analysis of self-boundary in psychotic experience within a sociocentric culture

**DOI:** 10.3389/fpsyt.2023.1215412

**Published:** 2023-07-20

**Authors:** Elizabeth Alphonsus, Lisa C. Fellin, Samuel Thoma, Laura Galbusera

**Affiliations:** ^1^Department of Human and Social Sciences, University of Bergamo, Bergamo, Italy; ^2^Department of Psychiatry and Psychotherapy, Brandenburg Medical School, Brandenburg, Germany

**Keywords:** schizophrenia, psychosis, IPA, minimal self, boundary loss, intersubjectivity, culture

## Abstract

**Introduction:**

In the tradition of phenomenological psychiatry, schizophrenia is described as a disturbance of the minimal self, i.e. the most basic form of self-awareness. This disturbance of the minimal self at the individual level is assumed to precede the intersubjective disturbances such as boundary weakening. However, the role of intersubjective disturbances in the emergence and recovery of schizophrenic experience still remains an open question. This phenomenological study focuses on how encounters with others shape self-experience during from psychosis by analyzing this process from the perspective of cultural differences, which in current research is especially under-researched. While most phenomenological accounts are based on first person-accounts from Western, individualist cultures where the self is conceived and experienced as separate to others, the present study qualitatively investigates psychotic experiences of patients from Jaffna, Sri Lanka.

**Method:**

Semi-structured interviews were conducted with three participants with a diagnosis of schizophrenia or first episode psychosis. The interviews were transcribed and analyzed using interpretative phenomenological analysis (IPA). Eight group experiential themes were identified across interviews.

**Results:**

The data suggest that intersubjective processes of boundary weakening such as invasiveness and hyperattunement may shape minimal self-experience and more specifically contribute to a mistrust of the own senses and to hyper-reflexivity. Interestingly, boundary weakening yields pervasive emotions and can be experienced as a threat to the whole social unit. On the one hand, the strengthening of self-other-boundary was achieved through opposition, closedness and withdrawal from others. On the other hand, this study suggests that the re-opening of self-other-boundaries in response to the crisis may help establish connectedness and may lead to recovery.

## Introduction

In this study we primarily draw on a phenomenological understanding of schizophrenia as a disturbance of the minimal self and of intersubjectivity (1–3).

Especially in current phenomenological approaches, the disturbance of the minimal self has been considered as a primary feature of the disorder (3). The minimal self or ipseity refers to the immediate and pre-reflectively given impression that we are the subject of our experiences, that it is us making those experiences without having to actively reflect upon it (3). Although the minimal self is conceived as an *a priori* of experience, it is also an experiential dimension that may vary in intensity and even become porous or get lost (4–6). Already Schneider (1950/1976) (5) for instance claimed that this constant feeling that experiences are *our* experiences – what he called “Meinhaftigkeit,” i.e., “mineness” of experience – may well fluctuate, may lessen in the case of perception and thought and grow in intensity with certain emotions [(5), p. 124]. This dynamic aspect of mineness comes especially to the fore with regards to our lived body: The minimal self has been ascribed an essentially bodily dimension in terms of a tacit sense of inhabiting or dwelling in one’s body, and a consequent awareness of one’s own body as a sensorimotor subject in the environment (1). This awareness and inhabiting of our own body which the German phenomenologist Schmitz (7) called “bodily self-sensing” (ger.: “eigenleibliches Spüren”) is especially dynamic with regards to bodily movements such as rhythms of breathing or heartbeat etc. and may also become fragile if one’s bodily integrity is dissolved (7, pp. 50–53). This particularly applies to persons with schizophrenia who report a weakening of ipseity – their sense that experience (and especially their bodily experience) is truly one’s own is diminished. Importantly, several empirical studies indicate a disruption of embodied processes in experiences of schizophrenia (8–11). For example, patients often report feeling that their bodily parts are disconnected from their body and cannot control them, they see doubles as external to their vantage point (autoscopic experiences), and report altered experiences of time (12). According to this perspective, persons with schizophrenia experience a lack or weakening of a sense of a stable and bounded self, especially at the most immediate and embodied level of experience. They fear being penetrated or engulfed by others and experience confusion regarding the contours or boundaries between self and the external world or others (13). Hur et al. (14) found that persons with schizophrenia were more likely to indicate a crisis of the minimal self, compared to controls. They reported an altered sense of body and ownership, sense of agency and self-reported subjective experiences, which were ascribed to an unstable minimal self (14).

Phenomenological studies of the experience of schizophrenia suggest that ostensible positive symptoms of schizophrenia such as auditory hallucinations and paranoid delusions reflect a weakening of separation of the self from the environment and others (3). Stanghellini (15) describes this loss of boundaries as a “pathological empathy and sense of openness,” which can lead to feeling like one is merging with the other and or losing the self [see also (16)].

The experience of a self-other boundary is tightly related to the sense of mineness. For example, phenomenological studies report desomatization (depersonalization) of the body during experiences of boundary loss: retaining the boundaries between the self and the outside world requires ipseity or a tacit sense of in-dwelling in the body (1). When mineness is weakened, the observing self becomes separated from the experiencing and bodily self. This means that the spontaneous act of sensing oneself in one’s body and boundaries is replaced by an ongoing reflective effort to maintain these boundaries and to interpret sensory stimuli in the third person. The result of a weakened minimal self consequently is a state of hyper-reflexivity where one’s actions are constantly observed and controlled from the outside. The sense of losing the contours of the self, watching the self as if from outside or fearing merging into others is seen as a consequence of weakened ipseity [(1, 3, 17–19); see also (20)].

Changes in ipseity or in-dwelling of the body thus may also contribute to confusion of ego boundaries between the self and other. Accordingly, Stanghellini and Ballerini (21) argue that when the minimal self is disrupted, one’s intersubjective experience is profoundly altered. Alterations in intersubjective experience such as invasiveness, emotional flooding, hyperattunement and hypoattunement are shown to shape and alter the person’s experience of the social world. This leads to difficulties in assessing others’ mental states, feeling invaded by others from without and within, and occasional feelings of merging with others (21). Also here, authors have investigated and shown how the self’s inner experiences and affection alter the experience of the social world, but they do not investigate how intersubjective experiences or self-other encounters may shape or alter the minimal self in return. In other words: we know a lot about the impact of self-affection (the self’s auto-affection) on the hetero-affection of the world and others but only little about the impact of the world and others on self-affection [*cf.* (22)]. We see this as a crucial gap in the phenomenological literature that still needs to be addressed and investigated.

Another shortcoming of phenomenological studies so far is that they do not take into account the significant role the cultural context plays in shaping schizophrenia emergence and outcomes (23, 24). According to Kulhara (25), consistent findings across various cross-cultural studies indicate that developing countries tend to have a larger proportion of patients (50-60%) with a good outcome and a lesser percentage with a worst outcome compared to developed countries at 2-year and 5-year follow-up. These differences persist at 15 years. Moreover, employment and social functioning outcomes in Global South countries are far superior (23, 26–29). Foremost, socio-cultural factors can impact the course of schizophrenia. In more collectivistic cultures, persons with major mental distress generally do not live on their own and may thus have greater access to a social network and support mechanism before and during crisis (26). For example, in a large sample of service users receiving public mental health treatment in the USA, Asian American and Latino consumers were considerably more likely than white service users to live with family members and to receive family support (30). We know from different studies that access to social networks is essential to recovery from schizophrenia. For instance, Degnan et al. (31) found that social networks are associated with symptomatic and functional outcomes in schizophrenia. On the therapeutic level, this is especially supported by approaches such as Open Dialog, which focus on strengthening the social network, and which have been proven particularly effective in reducing schizophrenia symptoms (32). In a nutshell, experiences of psychosis are shaped by the presence and quality of the social network.

Hence for phenomenological research, it is worth investigating how intersubjective and cultural experiences might shape subjective processes, even at the minimal level of the self. The present study tries to tackle this issue by focusing on the loss and recovery of self-boundary in a non-Western cultural context. Although the study is limited to culturally specific single cases its results can enrich the general concepts of selfhood and boundaries – an idea that is well established in phenomenological psychiatry where for instance Roland Kuhn claimed that “with every schizophrenic psychopathology starts anew and leads somewhere else” (33, p. 147; see also 34, p. 48). The study’s primary task thus is to explore (minimal) self-experience and intersubjective experience (of close relationships) during psychotic crises in a specific cultural (local) context, in order to extend our general understanding of how the presence of and the relation to others might shape and influence experiences of boundary disruption and of recovery of a boundary. It analyses how this relation to others leads to alterations in minimal self-experience and in what specific ways it might contribute to a strengthening or weakening of boundaries. We explore the phenomenon of boundary loss as a relational one, which describes on the one hand the boundary between the self and the external world, and on the other hand, the boundary between self and others (intersubjective level). We thus investigate how close relationships might alter co-occurring and inter-related experiences of boundary disruption and mineness.

Secondly, this study is one of the few in-depth phenomenological investigations focusing on the experience of persons from collectivistic or sociocentric cultures (35, 36) where the self is defined as including others. Moreover, Markus and Kitayama’s work (1991) (36) on cultural construals of the self maintains that persons from sociocentric cultures experience the self differently and this has significant implications for how we construct emotion, cognition, and behavior. They suggest the self in collectivist cultures is interdependent with the surrounding context and defined through relationships with others (self in relation to others).

By exploring the experience of schizophrenia, in its subjective and intersubjective dimensions, in a collectivist cultural framework, we aim at tentatively expanding and perhaps even challenging classical phenomenological assumptions reflecting more egocentric or individualistic cultures.

## Methods

We conducted an Interpretative Phenomenological Analysis (IPA) study with three male participants with schizophrenia or other psychotic spectrum disorders recruited at hospitals in Jaffna, Sri Lanka (please see Table 1 for participants’ demographic information). Potential participants were selected by the psychiatrist among those considered not to be in an acutely psychotic state, although they did show some positive symptoms. Recruiting participants proved very challenging. Many potential participants refused to take part in the study for various reasons (See Authors, in preparation).

**Table 1:** Research participants’ demographic information.

Participant nickname	Deva	Siva	Ranjan
Age	26	31	26
Gender	M	M	M
Job	Mason	Unemployed? Unclear.	Farmer
Reported Family Members	Mother, father, two older brothers, one younger brother, two older sisters, Dino (a cousin?)	Mother, father, a sister with whom he comes to the hospital, other younger brothers and sisters.	Mother, father and two younger sisters.
Whom do they live with	Parents	Parents	Parents
Inpatient/outpatient	Inpatient	Outpatient	Outpatient
Diagnosis	Admitted to hospital after psychotic episode. Suspected schizophrenia diagnosis.	Schizophrenia, unspecified	Schizophrenia, unspecified

Interviews were semi-structured and focused on four main areas:

1. exploration of relational life (e.g., could you tell me about the important people in your life?)
2. exploration of self-boundary (with a focus on self-experience and minimal self-experience) during psychotic crisis (e.g., could you tell me about a time you experienced great difficulty or turmoil^1^? Have you ever felt like you could not tell where you ended and others began?)
3. exploration of experience of close relations in these moments (e.g., were you aware of other people around you during the crisis? What was being around other people during a crisis like for you?)
4. How and which social relations hindered or supported the sense of boundary and ipseity (e.g., how did being around other people impact you during the crisis?)

Following inductive principles of phenomenological research, experiences of close relationships during boundary loss were openly explored during the interview. Follow-up questions generally focused on a deeper understanding of the participant’s experience of boundary weakening and self-experience, rather than probed for specific information. Research questions were kept at the back of the mind of the interviewer although they did not propel the discussion forward, thus we employed an inductive deductive approach to interviewing. Interviews were done by the first author and effected in Tamil, the language spoken in Jaffna, Sri Lanka. Each interview was between 45 and 80 min long. The Tamil recordings were translated and transcribed into written English. Ambiguities in translation and the use of idioms were discussed with native speakers and a Tamil language teacher. As translations are always influenced by the translator’s choice of words and syntax, this alters the participant’s account (37). Linguistic issues were taken into consideration in the coding process and integrated in the “linguistic comments.” In IPA the transcripts can be seen as a product of shared meaning making between participant and researcher, and reflect the researcher’s understanding of the participant’s own sense-making expressed through words (double hermeneutics). The linguistic issue thus added a layer of interpretation, which was taken into consideration since the early stages of the analysis.

## Analytic process

Each interview was analyzed individually following the IPA method (38). IPA is a qualitative phenomenological method that allows an in-depth exploration of narrative material. It aims at extrapolating the central meaningful themes from a text and is based on several coding steps through which the process of interpretation (double hermeneutic) unfolds. The first step of IPA is the production of exploratory comments, which include descriptive, linguistic, and conceptual comments (38). In a subsequent step, comments are brought together into experiential statements, which are later on grouped and clustered in personal experiential themes for each interview as described by Smith et al. (38). These are finally brought together across interviews in group experiential themes and the relations between themes were conceptualized into a dynamic model. In the final stage of the analysis, themes are brought together across interviews in overarching personal experiential themes, which are finally further clustered in group experiential themes for all data. The analysis of the first interview was cross-checked by an expert in IPA (LG) before proceeding to the analysis of the other two interviews. The audit trail of the analysis was checked by LG and LCF. This process ensured the trustworthiness of the analysis. Investigator triangulation also improved the depth of our analysis: by focusing on and discussing comments and themes at all stages and by engaging in recursive analysis loops we could deepen our interpretative account and at the same time ensure its reliance on the participants words (credibility).

## Reflexivity

The interviewer grew up in the Northern Province of Sri Lanka where these interviews took place. Hence, the interview’s shared Tamil identity and culture, and their ability to speak Tamil meant that it was easier to connect with participants and validate their experiences. Moreover, the interviewer was able to understand many cultural references, such as pressure to get married to be accepted by one’s community, or the emphasis placed on having a good reputation or face in one’s community.

However, being from a different religious community within a shared geographical and cultural background shaped the process of data generation. Due to the interviewer’s Christian cultural background, she had a limited understanding of Hindu cultural models of psychological distress and healing. During the interview, the researcher had difficulties understanding the meaning of specific rituals such as the dissolving of the charm. Besides, the interview process was influenced by the interviewer’s anxieties around correctly comprehending participants and being able to effectively communicate with participants in Tamil since she does not currently use Tamil on a daily basis. As a result of this anxiety, the interviewer sometimes focused on understanding exactly what the participant was saying, rather than freely exploring the participants’ experiences as they emerged in the “here and now.”

## Ethics

This study complies with the ethical guidelines of World Medical Association’s Declaration of Helsinki (2013) (39) for conducting medical research involving human subjects. Following the Helsinki declaration measures were taken to ensure the wellbeing of participants and minimize risks. Participants were recruited by medical doctors and the psychiatric nurse and were thus recruited by qualified healthcare professionals. However, doing research with participants who experienced psychosis and hospitalization is highly sensitive and ethically complex. As discussed in a forthcoming paper, key ethical concerns involved navigating the tension between protecting participants from harm, ensuring their freedom of choice and recognizing their capacity for agency, their right to self-expression [see also (8)]. To avoid coercion, several steps were hence taken to ensure freedom of consent and safety, given our commitment to facilitating their ability to articulate and make meaning of their own experiences (Authors, in prep). We conceptualize consent as a process, and so sought affirmative engagement throughout the research process, checking in with participants before, throughout and after each research contact, to ensure that they were free and content to continue to participate. Information sheets were written in clear, understandable and jargon-free language and attention was paid to presenting this information to ensure that they fully understood what their participation in the study would involve (e.g., what terms meant; how their interview data would be used…). Prior to gaining verbal and written consent, we ensured that all participants were fully informed of the purpose and focus of the interviews and of their rights to withdraw and omit questions by checking with them what they understood about their ethical rights and protections. The interviewer ensured potential participants were fully informed about the independence and confidentiality of the research from their hospital care, making it clear that she was an independent researcher, to maintain a level of separation between the hospital and the research team. The interviewers were flexible in their interactions and adapted phrasing, the form of questions and style of interaction to the participants’ needs. Following the interview, the researcher checked with people how they had experienced the interview. The interviewer is a trained professional and worked with the hospital staff to ensure that participants were in safe situations at the time of the interview, and that if distressed afterwards, there was support in place for them. All participant names have been changed to ensure anonymity.

Participants were informed of anticipated risks and benefits. They were informed of the difficulty of sharing psychotic experiences, limits to confidentiality and were given the researcher’s contact information. The medical staff and the researcher assessed risks such as acute psychosis before starting the interview by ensuring they were able to answer basic questions and were able to give consent. All participants of this study signed the informed consent form before taking part in the interview.

## Findings

We identified 9 intertwined personal experiential themes that encompass all three interviews (38). These 9 themes were then further grouped into 3 group experiential themes (see Table 2). First, in our participants’ experiences, boundary weakening seemed to yield hyper-reflexivity and diminished ipseity. Secondly, boundary weakening was experienced as a pervasive threat to both the self and others. Finally, attempts to re-establish self-boundary were characterized by both closing and opening up the boundaries of the self to others. In what follows, we present the three categories with the respective group experiential themes.

**Table 2:** Themes.

**1**
**Group Experiential Theme 1: from boundary loss to ipseity disturbance**
1.1
Feeling invaded by others and by external entities*Ranjan: There is a person inside my body. His hands and his legs are inside (p. 12)Deva: Stories, they tell scary stories through my mind (p. 2)Siva: I saw that girl. I looked straight at her…They are going to beat me up (p. 20)*
1.2
Altered experience of the self and body*Ranjan: If you check, you will see that my spinal cord has been taken… it has melted (p. 28)Deva: I get a little dizzy. It feels different/strange (p. 10)Siva: My healthy body has been muffled/suppressed by them (p. 15)*
1.3
The self and body as out of the participant's control*Ranjan: Please go away from my heart, please go! [touches his heart] (p. 33)Deva: If [I] take something, it’s like they are making [me] take it…If I pour wateror if I wash rice with my hands, it happens very quickly. That's his control/power (p. 10)*
1.4
Own experience as untrustworthy*Ranjan: We can’t see it [Mother Jesus] with our eyes. Only spiritual eyes can see it (p. 7)Deva: Arun doesn’t come and do it. Arun does it from the place he is in…from his house, he is doing it (p. 5)*
**2**
**Group Experiential Theme 2: Boundary loss yields fear and is a pervasive threat to self and others**
2.1
Fear: violation of boundary as a threat to the self*Siva: I go into an angry trance/lost self-control, I hit them (p. 180)Deva: When the fear goes and goes. My whole body shivers (p. 30)Ranjan: Like something fell, I was hit… I was afraid (p. 37)*
2.2
Violation of boundary threatens the social unit*Deva: They [Arun’s family] make us [the family] get angry with each other. The reason for all of this is Arun (p. 4)Siva: They [other entities] make me strike my heart and shout. My dad… They disgrace my father sometimes (p. 48)*
2.3
Being a self through the eyes of others: the importance of social recognition and belonging*Siva: P: They [villagers] say "the way you once were…look at what has become of you. Just stay this way and be. Don't come to the village" (p. 3)Ranjan: When I think about that, I can’t tell [anyone] about it (p. 9)*
**3**
**Group Experiential Theme 3: Re-establishing self-boundary through intersubjective closedness and openness**
3.1
Closing up to others during boundary-loss as an attempt to strengthen the self-boundary*Siva: He [the priest] spat. I didn't spit. I didn’t clear my throat and spit (p. 25)Deva: P: My mind feels sick [when I’m with others] (p. 22)*
3.2
Recovery through opening up to others and external entities*Siva: They said my name, they enunciated [my name], they dissolved it in the sea. They let my bodily pain loose (p. 27).Ranjan: Me and the spiritual man, we can live together happily. The spiritual man is in me, the man in my body isn't giving me trouble (p. 37)*

### Group experiential theme 1: from boundary loss to ipseity disturbance

**Figure 1:**
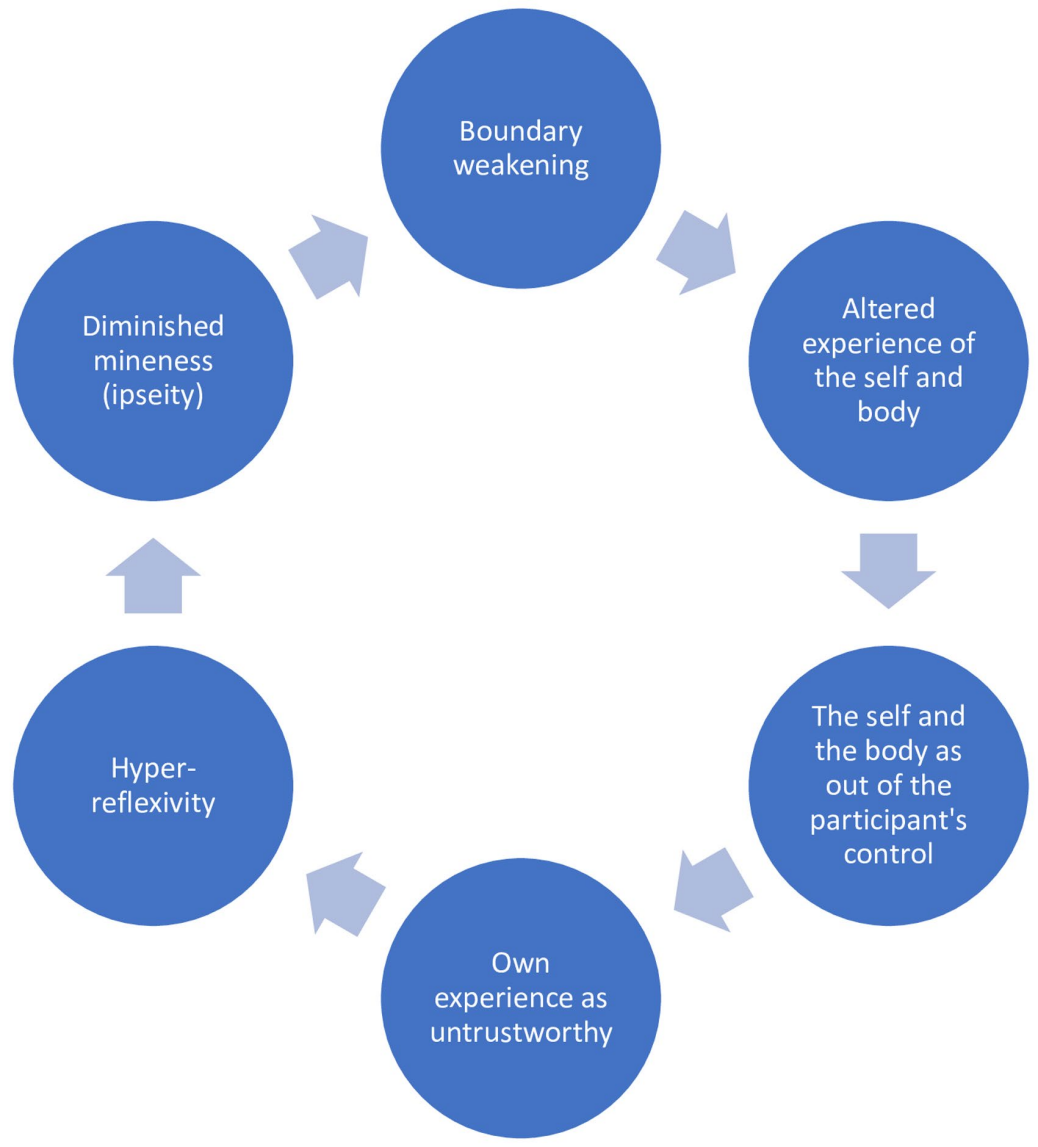
Group experiential theme 1: from boundary loss to ipseity disturbance.

The first 4 themes can be seen as a set of interrelated processes (Figure 1). During psychotic crises, participants experienced their body as being invaded by external entities (boundary loss). These external entities include neighbors, spirits and a god-like person. For example, Ranjan states “the man inside me. The man is made of skin (p. 12).” When there are others in the body, the self is experienced as fragmented. The fragmentation of the self contributes to altered experiences of the self and parts of the body. When participants experienced alterations in bodily experiences, they also experienced their own selves and bodies as out of their control. Siva reported feeling “wild (mad, uncontrolled, fanatic, violent or manic) (p. 17).” Deva reported that his “nice thoughts” would disappear due to the power of his neighbor, Arun (p. 16). Finally, this feeling was related to a sense of untrustworthiness toward one’s immediate and embodied experience (ipseity disturbance).

#### Feeling invaded by others and by external entities

Participants felt that other persons or entities had disrupted the boundaries of the self and their body. This was related to a feeling of fragmentation, and in some cases, a lack of a clear identity.

According to Ranjan, an external person had come to live in a part of his body. He describes the man inside him as “just like me” and being “together with my hands and legs.” According to Siva, a spirit inhabited his body in the form of a charm, and the presence of the spirit caused “unwanted (or unnecessary) thoughts,” such as the desire to take care of stray animals. Deva described his neighbor as “telling scary stories through my mind.” All these experiences refer to a weakening or loss of boundary of the self to the external world and to others. Ranjan experiences the weakening of the boundaries of the self as a sense of being occupied by a spiritual man and other gods in his body:

P: There is a man inside me [the spiritual man]. Because of this man, these fights started. Because this man doesn’t know how to get along [with others]. Remember how I told you the god said he had blessed me; he had blessed me. Due to a small problem, one day he [the spiritual man] threw a jug and scolded [another god in his body]. He talks from inside me. He’s inside (p. 12).

#### Altered experience of the self and body

Participants describe altered physical states, diminished physical awareness or the inaccessibility of particular bodily states as being related to and following the experience of being influenced by others and external entities (such as gods) on the body and the self. Moreover, parts of the body were felt as no longer being directly accessible and controllable by the participants.^2^

P: Sometimes if it’s something good, he'll [Arun] take it for himself. He makes you forget them.

I: I don’t understand. Can you say that again?

P: When I think nice thoughts.

I: Yeah.

P: He takes them for himself.

(…)

I: How are you after that?

P: I get a little dizzy. It feels different/strange.

I: It feels different/strange? How?

P: It feels different/strange. A stiffness/coldness/frigidness (p. 10).

Deva does not have direct access to some of his thoughts, as his neighbor has made him “forget them” (p. 10). The removal of “nice thoughts” by the neighbor leads to altered or “different (or strange)” physical states such as a “stiffness or coldness” and dizziness. He later reports shivering due to his neighbor, who attempts to manipulate his thoughts. Here, the loss of intersubjective boundaries with his neighbor Arun seems to alter Deva’s bodily experiences.

Ranjan experiences the body as permeable when other entities can attack his body from within: his heart “felt like meat” when Father Jesus struck his stomach from within his body (p. 54). He states that he finds it difficult to breathe because the gods press his chest bones and torment him (p. 40). Thus, violence of other entities alters Ranjan’s awareness and control of his body. Arts of the body are experienced as though they were external entities as he cannot feel them or control them and this is clearly linked to the gods’ violence. Ranjan states that his ears have been ruined by the spiritual man and he can no longer “hear” the entities in his body (p. 41).

Participants showed diminished bodily awareness and reported feeling that their body was experienced as weak, fainting or even dead. Other persons (his neighbor, and other people) attempt to influence Deva by saying his name. Deva then describes a sense of “tiredness” “darkness” and a “fainting” sensation which suddenly comes over him:

P: I think about it in my mind over and over again. I fear. "Deva. Don't let him lie down. Deva, don't let him sleep" They are trying to force me without my consent and kill me. Due to all of this I'm afraid.

I: Fear comes. What fear? That you won't be able to sleep?

P: Um.

I: Why is there fear? You don't know? So what if they say things?

P: Fear. They'll use my name. Using my name and looking at the number of letters. [spells his name] one…two… four…five. There are three letters. What thing is this? [the last letter of his name] Three letters. They want to show their power to [says his name]. My mind. My heartbeat is going to fall.

I: Yeah. It goes into your heart?

P: Yeah.

I: What goes in?

P: Fear goes into my heart.

I: Fear goes.

P: When the fear goes and goes. My whole body shivers.

I: Yeah.

P: I become afraid.

I: Yeah.

P: A little sort of tiredness comes over me. A little.

I: Uhum, uhum.

P: Then fainting like fainting. A darkness. A fear that's like a sort of darkness (p. 30).

The participant’s lack of energy, feelings of “fainting” and “darkness” may point toward a diminishing bodily awareness. These feelings of “fainting” and “darkness” may indicate an inability to perceive his inner bodily states. Similarly, Ranjan reports that his spinal cord has “melted” and all his bones have been removed by the gods (p. 6).

P: God. Like God's talking. A very powerful God. They [neutral form] have taken out my spinal cord.

This description is also suggestive of an altered perception of his body experienced, as linked to being “invaded” by others. This weakened sense of inhabiting the body makes it difficult for him to perform simple actions like to wash his face and brush his teeth.

Ranjan mourns that his masculinity or virility has been spoilt or ruined by the gods, which too alludes to altered physical perception and a weakening sense of inhabiting the body (p. 13).

#### The self and body as out of the participant’s control

Participants experienced a loss of control over their own behavior and thoughts. This loss of control was sequentially described as following the experienced ability of external entities to alter or diminish their physical perception. One might here suggest, that as a result of the participant’s loss of control, the self is no longer experienced as having independent agency. Moreover, from the participants’ narratives emerges that the loss of control over thoughts and behavior further enhances existing feelings of distance and dissociation from the body.

Deva and Ranjan felt that other entities controlled their inner thoughts and behavior, and that this permeability of the self-contributed to a diminished sense of agency and autonomy. For instance, other entities speed up Deva’s actions:

P: If [I] take something, it's like they are making [me] take it.

I: Yeah.

P: If [I] open a lid, it’s like [they] have to open it immediately. Or [the lid] has been placed somewhere quickly. If [I] take a basin or something like that, it’s like [they] have to take it quickly and [they] have put it back quickly. If I pour water or if I wash rice with my hands, it happens very quickly. That's his control or power (p. 16).

Deva’s intentions and actions overlap with the actions and motivations of external entities: the source or subject of the participant’s actions is ambiguous. The self as a source of independent agency, control and action was compromised by a sense that an external entity is controlling.

A weakened sense of inhabiting the body makes it difficult for Ranjan to perform simple actions like washing himself. His repeated use of the words “cannot” suggests a helplessness due to a loss of control over the body to other entities, and the difficulty of conveying his experience to others.

P: I cannot brush my teeth, I cannot wash my face. I cannot tell my mum, I cannot tell anyone. I am feeling sad.

#### Own experience as untrustworthy

Participants described how they relied on knowledge gained through the presence of external entities and thus disregarded their own immediate sensory experiences. The permeability of the self to external entities thus seems to contribute to the distrust of the participant’s sensory experiences. Mistrust of senses creates a sense that things are not as they appear to be, and plays a role in increased reliance on reflection. The mistrust of the senses seemed to be linked to boundary weakening.

For example, as seen in the quote below, Ranjan mistrusted and disregarded his own perceptions, and believed that he perceived the external world through the eyes of other entities such as Mother Jesus. He distrusted knowledge gained through sensory input (e.g., the participant’s own eyesight). In a vicious circle, it seems that the more participants questioned and distrusted their immediate perceptions, the more they relied on the intrusions of thoughts and perception experienced as belonging to others or external entities to make sense of their environment. Ranjan states:

P: I told my mum “They’ve [Mother Jesus] taken out my spinal cord”

I: Yeah.

P: She didn’t listen to me. We can’t see it [Mother Jesus] with our eyes. Only spiritual eyes can see it.

I: Only spiritual eyes can see it?

P: I can’t see it. I can only hear it speaking (p. 7).

(…)

At the same time the spiritual man was in my body. So, when I was going to work. The spiritual man has the eyes to see him. Spiritual eyes (p. 18).

From these quotes, it emerges that, when the spiritual man is present in his body, Ranjan relies on “spiritual eyes” to make sense of the world and understand what is going on instead of relying on his own perception or eye sight.

In Ranjan’s words, one can also notice a contradiction between what he sees and what he hears. He explains that he cannot see the god, but he can hear the god speaking. As a result of the influence of an external entity (the spiritual man), he seems to develop a greater distrust of his sense of sight, and an increased reliance on his sense of hearing. In the absence of visual stimuli, the participant makes sense of the presence of voices only he can hear (auditory hallucinations) by stating that “only spiritual eyes can see” the gods. He thus compensates the mistrust of his visual perception through a constructed belief that the external entity (the spiritual man) would see for him. This also highlights a tendency to distance himself from his immediate perception and to compensate with reflexive thinking and constructed beliefs: instead of “I see,” “I believe that an entity within me sees.”

Reliance on external entities combined with a deep-seated mistrust of the participant’s own senses contributes to the participant’s belief that things are not as they appear to be. Deva for instance distrusts his own direct perception of his environment, and relies on beliefs regarding his neighbor’s powers of psychokinesis. He thereby attributes hidden meanings to other’s behavior. For example, he states that his neighbor causes his father and mother to walk. He also blames his neighbor for other events in his home such as a change in water pressure; there is some hidden intentionality behind seemingly insignificant events.

P: Now, when my dad walks, he causes it.

I: Yeah.

P: When my mum walks, he is the cause of it. If you open the door, he shows his presence by opening the door. I just close the door, he closes it tightly. Um… The water. It is as though he is doing it, he makes the water run faster (p. 4).

Deva can see that his neighbor is not at home, and yet he believes that he is able to influence events in his house with his powers and to cause conflict between family members (p. 6). Blaming his neighbor for conflicts at home suggests that the participant does not rely on immediate sensory perception and experiences, and instead attributes hidden meanings and intentionality to understand the conflicts in his home.

Finally, the felt presence of others within the self was related to an experienced disconnection from one’s own thoughts, which also acquire an object-like quality. For example, in an already mentioned quote by Deva (see above p. 14), his thoughts take on an object-like quality, as he describes them as something that can be taken away or stolen. Thoughts are experienced as something that can be removed and “kept” with someone else, without a clear connection to the source of the thought (the person or body).

By feeling invaded by others, participants distrusted their own perception and thoughts. This distrust led to increased reflection to make sense of the world. Over-reliance on reflection was thus related to diminished spontaneity which – as described above, in a kind of vicious cycle – reinforced the participants’ perception that the self was occupied by others.

### Group experiential theme 2: boundary loss yields fear and is a pervasive threat to self and others

During the experience of boundary weakening, the boundaries of the self, the boundaries of the family unit and the relationships between family members were experienced by participants as threatened. This was related to fear and sometimes even anger. Another central aspect was that participants highlighted the importance of belonging, and it was interesting to notice how the self as seen by the community, or the social self, often took precedence over how participants viewed themselves.

#### Fear: violation of boundary as a threat to the self

The weakening of the boundaries of the self-stirred an intense fear, which sometimes turned into anger against the self and others.

Deva describes the experience of being controlled by someone external (thus losing or weakening the sense of his boundary and very identity), which provokes intense fear. This fear is stated explicitly and reported through bodily states such as “shivering.” The repetition “goes and goes” suggests the intensity and continuity of the participant’s fear. “Whole body” suggests that the power of the voices is experienced as a threat to the body and that boundary weakening is a physical experience. The experience of his “heartbeat falling” is also suggestive of fear related to a weakened sense of self. The participant’s fears are compared to” a sort of darkness.” The simile of darkness and fainting suggest being unable to see or know something. As outlined in the previous theme, the loss or weakening of the self-boundary seems to lead to a sense of disconnection from his physical states, which is here conveyed through the simile of darkness and fainting. Thus, fear during boundary weakening is related to a sense of “darkness” or not-knowing regarding the participant’s inner states. Fear was described as a sense of threat, of being endangered by other persons, which again was related to a sense of being invaded or losing the self-boundary. This can be exemplified in Siva’s account:

P: Not afraid. But I'm scared you'll do something to me, that fear is there.

I: Yeah. So, you feel people might do something to you? Me or someone else you speak to? That they'll do something to you?

P: Now I don't think like that now. But suddenly this fear comes. One minute…five…It comes for a bit (p. 35)

(…)

P: Before I get into fights whenever someone said something. No matter how many people came, they couldn’t catch me. I got into an angry trance/lost self-control, I hit them. When they bring me to hospital, I beat them up. I got into an angry trance/ lost self-control, and I hit them.

I: Why do you beat them up? Cause you are afraid they'll come beat you up?

P: No… they also beat me up. I beat them up. Because they'll take me to hospital. They’ll take me to hospital (p. 18).

Here it becomes clear how anger and even violence stem from fear and are a reactive response to an experienced threat to oneself. Siva fears that others may do something to him or take him to hospital. This fear is transformed into anger, and violence is used to defend and demarcate his own boundary.

#### Violation of boundary threatens the social unit

The sense of being threatened by other persons or external entities that violate boundaries seemed to not only apply to the self, but also to the feeling that boundaries of the home have been violated, thus threatening the integrity of the social network that is the family. Deva states:

He [Arun] has a daughter. I can’t handle her atrocities/ostentatious displays. *unclear* She imagines marrying me. As soon as she marries me, it's like she'll stay in our house. Then it's like she goes back to her house. Then it's like she helps my parents. Then it's like she leaves that and goes back to her house. When I go to work, it's like I'm at her mercy. As soon as my father comes… as soon as my father comes… she gives him his sarong [a type of garment worn by men]. She laughs. As soon as I come home, if I call her. It’s like she asks me why, fights with me and tries to cut me. She ruins our house like that, she may destroy it. The powers of Arun and his family are like that (…). They're games are what's happening in our house. Moreover, they scare my mum and my dad (p. 4).

P: It's a [great] fear. Fear comes like an iron rod. They make us get angry with each other. The reason for all of this is Arun.

I: Yeah, yeah. [You] get angry with others?

P: Yeah.

I: Your house members, they fight with each other. Are you saying Arun came and did it?

P: Arun doesn’t come and do it. Arun does it from the place he was in [i.e. Arun's home]

R:Yeah.

P: From his house, he is doing it (p. 4).

In Deva’s experience, his neighbor Arun can influence the relationship between family members by causing fights between them. Intersubjective boundary weakening (where Deva feels that Arun can affect his and his families’ mental states from his home through incantation or the virus) is seen as causing division and the disruption of the relationships within family members or as causing intrafamily conflict. Moreover, Arun’s powers of incantation over the family home (highlighted in the sentence “from his house, he is doing it”) represents a threat to the relationship between family members.

Not only does Arun’s daughter threaten to ruin Deva’s home, Arun’s daughter threatens to violate the boundaries of the family home by attempting to marry the participant, slowly becoming a part of his family by assisting Deva’s father. More importantly, Deva thinks that Arun’s daughter will move into their family home if he marries her. Hence, Arun’s daughter’s “atrocities/ostentatious display” represents a threat to the boundaries of the family home, and the participant’s position within the family. The participant’s experience of intersubjective boundary weakening (through Arun’s powers of incantation/psychokinesis) is experienced as a conflict between two families: Arun and his daughter’s games threaten the integrity of Deva’s family and cause divisions in Deva’s family. The word “house” can refer to the shared physical space between family members as well as an intricate network of family relationships.

Deva’s experience of Arun’s incantation/virus suggests the loss of intersubjective boundaries with his neighbor is also experienced as a threat to the social network he belongs to, not just the individual. Ranjan also experiences boundary weakening as a threat to his social network. External entities (the semi-gods) threaten the participant, but also his family. By tormenting family members, the gods target the individual as well as members of the social network he belongs to. This suggests the participant experiences not only a threat to the self but a threat to his family members:

P: Make me scream. Now they are saying: "come home." They make me strike my heart and shout. My dad… They disgrace my father sometimes. When they disgrace him [my father] nothing can be done (p. 48).

Moreover, Ranjan suggests the gods’ violation of his self-boundaries also threaten Ranjan’s father’s reputation. The use of the possessive pronoun in “my heart” suggests boundary weakening is experienced as undermining the individual self. However, the gods’ actions also “disgrace” the father. The use of the word “disgrace” suggests that self-boundary weakening contributes to Ranjan’s father’s loss of face. The notion of a loss of face suggests that boundary weakening threatens the participant’s family, and his father’s reputation or position within the extended family and the broader community. Ranjan’s experiences too point toward the notion that boundary weakening can be experienced as a threat to the social network.

#### Being a self through the eyes of others: the importance of social recognition and belonging

In the last two themes it became clear how the experience of losing or feeling violated in one’s own boundary is pervasive of both the self and the social environment. The current theme emphasizes the intertwinement of these two levels (self and social) in the participants’ accounts and more specifically the importance of social belonging for the self.

Throughout these accounts a pattern of narrating about oneself through the eyes of others emerged. When talking about themselves, participants often reported about the perspective or relevant others on themselves instead of expressing their own. Moreover, participants recalled crises at times when there was dissonance between their own view of themselves and others’ views. In contrast, when participants felt good, they talked of a congruence between their and others’ perspectives:

P: It feels like I'm good. When I'm in the state of mind, I feel that I do what is right. I am good. Only others can see that he [the participant] is like this.”^3^

This congruence suggests that other’s perspective may contribute and shape the participant’s narratives of the self during recovery. When talking about his mental health Siva uses the expressions “going about well” vs. “going about badly [in the village/community]” and “going about with an unkept appearance,” which highlight the significance of the how one goes about in the presence of the community, and how one is seen by others, i.e., their social self or public identity. It suggests the participant’s tendency to construct their experience in terms of how others see them. Siva even defines recovery in terms of how others see them:

From my point of view, I was going about quite badly for two months. When I was in the village, the people in my village would think and talk about it. Now I'm good. I say, "I will keep her well [take care of her well]" and I need to look for and marry a girl.

Siva defines “going about badly” in terms of a loss of reputation in his community as he remembers people talking about him. Moreover, the contrast between “I was going about badly” and “now I’m good” suggests the significance of the change. Acts such as marriage, which emphasize social acceptance and connectedness to the community, are seen as signs of “going about well.” Siva appears to structure his narrative around what others in his community say about him, rather than how he sees himself. This becomes evident throughout the narrative as he constantly talks about the events of the psychotic crisis and recovery in terms of what others said about him. Even when the interviewer attempted to prompt him into describing how he felt about the events, Siva continued to describe other’s perspectives on his situation. This might suggest the significance of others’ perception of him, and the relative insignificance of his own perspective of the crisis. Importantly, in the following quote, we see that social exclusion or community rejection becomes a lens through which he experiences the psychotic crisis. For Siva, it appears that “the crisis” or “the problem” is known, understood, and experienced through the changed way in which the community sees him during the crisis.

P: Cause before I was good. When there was a celebration or something…

I: Yeah. Yeah.

P: During the celebration, I used to be there. Helping people and doing things.

I: You were there and you helped?

P: I would be there to help. I helped. That's why when some people see [me] they cry. My relatives, even my distant relatives,

I: Yeah…

P: They say "the way you once were… look at what has become of you. Just stay this way and be. Don't come to the village."

### Group experiential theme 3: re-establishing self-boundary through intersubjective closedness and openness

In this last category, we summarize participants’ experience of relations with other persons during moments of boundary loss. Participants generally described the presence of others as being difficult to bear. During moments of boundary disturbance they often tended to oppose others or withdraw from social relationships. This might be arguably seen as an attempt to strengthen and feel their boundary and avoid the feeling of being invaded or overwhelmed. However, in other accounts participants reacted to boundary loss in the opposite way: by opening up and letting themselves be influenced by others or by external entities. These moments were especially related to the recovery process.

#### Closing up to others during boundary-loss as an attempt to strengthen the self-boundary

During the crisis, participants disengaged and distanced themselves from relationships and did not allow others to influence them or their decisions. Participants also tried to strengthen interpersonal boundaries by positioning themselves in opposition to others; hence they were able to affirm their separateness.

In the following quote, Siva refuses to spit on a chicken during a ritual to release a bad spirit within him because he is against hurting animals:

P: I thought "poor chicken. They are killing it"

I: Yeah.

P: That's why I didn't spit on it. You shouldn’t spit on a dead person.

I: yeah, yeah. So, when they were doing this thing, this chant, you kept thinking about the chicken?

P: Not about the chicken. I was listening to their chant and watching them kill the chicken. He [*the priest*] spat. I didn't spit. I didn’t clear my throat and spit (p. 25).

Diminished attunement and increased tension between the participant and the community’s beliefs is highlighted in how Siva contrasts his own behavior against the priest’s: “he spat. I did not spit.” Separateness from his community is further emphasized when Siva openly reproaches his community: “Can you spit on someone who has just died?” His values appear to conflict with those of his community, and Siva resists taking part in the ritual while standing up for his own values. By positioning himself in opposition to his community Siva might assert and strengthen his own identity boundary.

Deva experiences difficulties being around others during moments of boundary weakening. When he experiences the power of his neighbor’s enchantment on his inner states and the boundaries of the self are compromised, he prefers being on his own, stating that he needs “to be away a little”:

P: Even when I'm at home I feel mad/restless.

I: You feel mad/restless?

P: Yeah.

I: Why does it feel mad/restless?

P: *unclear*

I: When you come home, won't other people be there? Is it good that people are with you at that moment? or is it quite difficult?

P: Usually it's quite difficult.

I: Is it quite difficult? Why?

P: My mind feels sick (p. 22).

Thus, when the participant experiences his boundaries as weakened (thus feeling the influence of the neighbor’s powers of enchantment), “his mind feels sick” and he finds relief by withdrawing from family relationships.

#### Recovery through opening up to others and external entities

In particular cases, allowing the influence of others or external entities instead of withdrawing or opposing them, was exactly what seemed to help strengthen their boundary. For instance, Siva takes part in a ritual which causes the disappearance of his “unwanted (or unnecessary) thoughts” concerning taking care of stray animals, socially constructed as a charm (lump) in his stomach, which is removed through the sacrifice of a chicken. While he affirms his skepticism concerning the ritual, the ritual which occurs outside his body in his experience causes the “bad spirit” to go away:

I: So, you think some good things happened to you because of this chant (or incantation)?

P: I don’t have those thoughts. I don’t think that I need to catch dogs. That I need to raise them. That thought isn’t there. I don’t bother people. If you come and inquire in my village, [you’ll] know (p. 25)

(…)

P: In the same way that if you eat sand, it becomes quite hard. The charm was like that. They massaged it with oil. They gave me herbs. So, it [*unnecessary thoughts and behaviours*] went.

I: So, if you do this, do this… So dissolving [ashes?] in the sea, that makes your unnecessary/unwanted thoughts go away.

P: That has all gone away.

I: All gone away. How?

(…)

P: Instead of me, for the evil spirit, for that evil Satan, they create it. It accepts the chicken as a sacrifice (p. 28).

(…)

P: It left 6 months after I went to the temple. For the last 4 months, I’m doing well and going about well. I’ve got a good reputation in my village (p. 31).

The participant previously got into trouble by burning the bodies of stray animals at his relatives’ home; after the ritual, he no longer engages in such behaviors. Hence, allowing or giving in to a blurring of boundaries (as an event outside his body is allowed to influence events within) contributes to freedom from the evil spirit.

Here the weakening of boundaries between the inner world and the outer world, helps remove the occupation of the participant’s body by a “bad spirit.” By allowing others to influence him and weakening interpersonal boundaries, the participant is able to restore his bodily boundaries which had been violated by the bad spirit. Paradoxically, this opening up of interpersonal boundaries helps re-establish the boundaries of the self, i.e., to stop the psychotic experience of being invaded by external entities.

One might hypothesize that, opening up to the community by letting himself be influenced by the ritual also means a restoration of the participant’s ties and – arguably – of the sense of belonging to or acceptance by the community (or family). The participant’s increased sense of belonging is evident when he asks others for “forgiveness” and states that he has now acquired a good reputation. Moreover, the disappearance of unwanted thoughts that his community disapproves of is seen as an indication of good health.

Although from a different perspective, the following quote also exemplifies the healing effect of opening up boundaries and establishing connection. Even if the following example does not concern an interpersonal situation, Ranjan talks about how relating and is accepting the external entity that threatened or disrupted his self-boundary lead to a more stable sense of self.

P: It felt like someone hit me with something very big….

….I: What did you do when you were hit?

P: I didn't do anything. I didn't get angry.

I: You didn’t get angry.

P: I was afraid. Now…

I: So, you didn’t get angry but you were afraid? You didn’t get angry but fear.

P: Fear.

I: Why? cause you were afraid that would hit you again?

P: Yeah. I was terrified then. They held me there.

I: How did you know you were afraid?

P: How fear normally comes. Before I was afraid. Now I just calmly tell them: "you go, we want to be in peace, the two of us will be in peace" But they aren't going. They are with us.

I: Who can live in peace?

P: Me and the spiritual man, we can live together happily. The spiritual man is in me, the man in my body isn't giving me trouble. I can happily go to the shop, eat. I can do everything. The spiritual person, the holy one, Mother Jesus and Father Jesus are there (p. 36–37).

Instead of protecting and closing up his boundary by fighting or rejecting the “spiritual man,” Ranjan engages and interacts with him and accepts him as a part of himself. In this sense also here we can see how the opening up of boundaries and giving in to external influence is –paradoxically – described as a way toward the recovery of the very self-boundary and of the sense of self.

## Discussion

### Violation and weakening of boundary may contribute to diminished ipseity and hyper-reflexivity

Within phenomenological literature on psychosis, the weakening of the boundaries of the self has been understood as a consequence of the loss of ipseity (1–3, 21). In contrast, our findings suggest that the weakening or loss of self-boundary is not a mere epiphenomenon of an ipseity disturbance, but might rather contribute or even lead to the very ipseity disturbance.

The suggestion is especially grounded in the first 3 group experiential themes, which are interconnected in a dynamic process that shows a chain of experiential alterations starting from the disruption or weakening of self-boundary and ending with the weakening or loss of mineness (Figure 1).

In a first step, the experienced violation of the self-boundary by external entities and persons altered the participants’ experience of their body. These alterations included changed or diminished embodiment, such as the feeling that their body was dead. Participants attributed these changes to the presence of other entities and persons, who trespassed the confines of their body and mind. Experience of others and the external world as invasive and overpowering diminished the sense of inhabiting the body and mind. This led to a sense of loss of control over their own body, a sense of being steered and controlled by others, which were described as even having control over their bodily states, perceptions and reactions. Thus, participants lost trust in their own perception and senses, losing a first-person perspective of their experience: they increasingly relied on (hyper-)reflexivity (thus experiencing themselves in the third person) and they lost the immediate and embodied access to their self (loss of mineness and ipseity). Boundary loss thus seemed to impede participants’ proximal or immediate experience of the world through their own body and require instead to rely exclusively on distal pole of experience (40).

In our model, it is particularly interesting to see the role of the experience of mistrust in one’s own senses as being related (and immediately preceding) the weakening of ipseity. Besides phenomenological accounts of schizophrenic experience that implicitly assume this role (3, 41), the relevance of trust and mistrust in one’s senses and its relation to the self has been an almost classic theme in phenomenological literature. Already Straus stressed that self-sensing and intermodal sensing of the world must always be seen as a unity for which he uses the term “sympathetic sensing” [(42), p. 373; see also von Weizsäcker 1950, p. 79; (43)]. This idea of “perceptual faith” into the world and its connection to self-affection has been re-elaborated in recent phenomenological accounts on schizophrenia (15, 44). Our study seems to corroborate these accounts by especially suggesting that persons with schizophrenia may also distrust knowledge gained through sensory experiences due to relational experiences associated with boundary weakening. We found that hyper-reflexivity and diminished spontaneity were experienced as being directly related to boundary weakening, thus further supporting the possibility that boundary weakening may impact and shape the minimal self. However, further research with larger and culturally different samples is needed to establish whether the mistrust of the senses is a common experience in schizophrenia and explore the nature of the relationship between boundary weakening and a mistrust of the senses.

Our findings highlight the importance of the phenomenon of boundary disruption as possibly playing a role in the emergence of disrupted ipseity. In the participants’ narratives we see how the disruption of the self-other boundary may alter other processes of the minimal self, such as auto-affection, (dis-)embodiment, the externalization of the body and (hyper-) reflexivity. Moreover, participants specifically spoke about the experience of being violated or invaded by others or by external entities, which might raise the question of whether the disruption of the self-other boundary might arise from overwhelming intersubjective situations such as traumatic events, or such as the pressure to conform with social norms and expectations, at the loss of personal agency and separate identity. These descriptions not only echoed precedent phenomenological analyses on the issue of boundaries for the emergence of psychosis (45, 46) but more specifically highlight the relevance of a phenomenologically grounded concept of traumatic experience especially for the case of psychosis as has recently been sketched out by several authors [(47, 48); see also (49, 50)]. Both our phenomenological study and these conceptual approaches point toward a possible directionality from the relational to the individual level. However, further studies are needed to further establish the complex relationship between self-boundary and ipseity both at the subjective and intersubjective levels, same as more phenomenological studies that explore, from a more general point of view, the manifold ways in which intersubjective experiences may alter the minimal self. These studies might shed light on the links between boundary weakening and other phenomenological experiences associated with a disruption of the minimal self such as morphological changes, somatic personalization, cenesthetic experiences and spatialization of body processes.

Caporusso (20) argues that experiences of boundary weakening or dissolution are not unique to psychosis, similar experiences can be seen in meditation, intimacy and art but questions whether they are part of the same unified phenomenon. In our view, in non-psychotic dissolution experiences, the disruption of self-boundaries is not associated with a loss of mine-ness, ipseity and hyper-reflexivity; it is something participants do to themselves or allow themselves to experience (See footnote 1). We see an openness to losing the self-world boundary and a willingness to let go of control over their experiences. Our participants experienced a loss of mine-ness, fear and a debilitating loss of control over the self and body. Hence, boundary disruption was something done to them by somebody else (heteroaffection).

Laing (51) maintained that the experience of “disembodiment” is a stable trait that is often correlated with “ontological insecurity” (being unsure of who one is or if one is real or alive and therefore when with others, fearing the loss of themselves/their identity). One example of this description of disembodiment is a feeling the body is separated from the self: Laing’s patients experienced threats to the body (e.g., mugging) as no “real harm.” According to Laing, the loss of the minimal self or the sense that one’s experiences are truly one’s own (or the “false self”) is a defence mechanism to cope with ontological anxiety. Moreover, for Laing, embodiment is a stable trait that some of us have more than others.

In our study we attempted to clarify the link between intersubjective experiences and disembodiment. While Laing’s descriptions of disembodiment reflected a mind–body duality, our participants also described altered bodily states and perceptual anomalies on the body caused by external entities. Moreover, while a phenomenological study such as ours cannot ascertain whether the loss of ipseity or disembodiment is more of a trait than a state, from a phenomenological vantage point our participants experienced altered bodily experiences as a consequence of the presence of external entities within the body. Hence, for our participants disembodiment, their ontological insecurity and loss of mineness was a state induced by others during a crisis.

### Pervasiveness of emotions during boundary weakening

We found that the experience of losing the boundary between self and others was characterized by intense emotional states including fear, anger and tiredness. A participant reported anger and fear as preceding paranoid beliefs that the interviewer might harm him. Sometimes, participants reported resorting to violence in response to fear and anger. Fear, conveyed through powerful metaphors such as “my heartbeat is going to fall” was especially experienced when the participant felt steered and controlled by other persons or external entities. These emotional experiences support theories such as Ciompi’s notion of *affect logic* and that emotions and cognition dynamically influence one another and that therefore a build-up of emotions within the system can lead to a loss of equilibrium and destabilization. According to Ciompi, destabilization caused by emotional build up can lead to reorganization of the system where delusional beliefs become the center of organization (52). Again, further research is needed to understand the complex relationship between emotions experienced during psychosis and their relationship to phenomenological processes such as boundary weakening, loss of ipseity and hyper-reflexivity.

### The role of culture in experiences of boundary weakening

Threats to the boundaries of the self were also experienced as a threat to the boundaries of the whole family unit. This is consistent with socio-constructionist and systemic theories on psychosis onset and maintenance within families (48), as well as with theories on sociocentric cultures (53, 35, 36) where the self is defined as including others. For example, threats to the self from external entities such as the gods or other persons such as Deva’s neighbor, undermined the boundaries of the self, the boundaries of other family members, intrafamily relationships and the boundaries of the home. External entities not only threaten the individual self but may also menace the social network, and the self as related to and connected with others. It however remains an open question if in sociocentric cultures, boundary weakening in schizophrenia may generally be experienced even more as a threat to the social unit, in addition to the individual self. This question needs to be further investigated. This question also relates to the bodily dimension: Lee’s study suggests there are cross-cultural differences in degrees of distress associated with bodily self-disturbances (54). While the weakening of the self-boundary is likely a universal experience (55), these results suggest there may be qualitative differences in how participants experience the weakening of these boundaries, for example increased tolerance of states of bodily dissociation and self-inconsistency due to a more flexible and changeable idea of the self-shaped by the social world (54). On the other hand, it is possible that experiences of the self as connected to others may have little to do with culture, but rather be a consequence of the very blurring of the self-boundary typical of schizophrenia. Clinical experience suggests that psychotic states are experienced as a threat to the social unit in non-sociocentric cultures as well. Nevertheless, specific ways in which sociocentric notions of the self and one’s relational world alter experiences of psychosis have only rarely been discussed in literature (56). Further and more extensive cross-cultural comparative studies are needed to explore the differences in the phenomenology of disruptions of the minimal self in sociocentric cultures and egocentric cultures and explore whether descriptions of the self as connected to others is merely a consequence of boundary weakening.

Our findings also lend support to the notion of the dialogical self where the self is experienced as having multiple perspectives (35, 57, 58). Kirmayer suggests that the narrative of the egocentric self is one of a strong individual self with an inner voice characterized by one perspective. In contrast, the narrative of the sociocentric self includes multiple voices and can be described as dialogical. Participants narrated their experience of the self often through the perspective of others. Siva spoke about himself and used the term “I” but usually in the context of how others saw them or “how he went about.” His narrative of the crisis and recovery was structured in terms of how his community members altered their perception of him. For Siva, recovery was primarily defined as a recovery of his reputation. Moreover, since boundary loss seemed to also pervade the social unit, the participants’ experience of the narrative self might include the perspective of their family and community members. Thus, participants’ narrative of the self suggests their experience of the self-included many perspectives (58). Also here, the question arises as to whether this is a typical characteristic of the narrative identity construction in collectivist cultures or if it applies to human nature in general. More cross-cultural qualitative studies are needed to explore cultural differences in participants’ construction of the narrative self during psychosis and after recovery.

### Self-boundary and social interactions

While participants often experienced boundary weakening as overwhelming and disruptive, there were also examples of positive experiences of boundary weakening where participants *allowed* other persons or external entities to permeate the boundaries of the self. These experiences were associated to recovery.

Siva sought to experience an opening of his boundaries to allow society and family members to influence experiences of the self. Interestingly, this experience of hyperattunement strengthened rather than diminished the self. Due to experiences of merging with others in his community, Siva’s unnecessary or unwanted thoughts (which caused him to behave in socially inappropriate ways) disappeared. While past literature has highlighted the need for separation during experiences of boundary weakening (59–62), our findings suggest that a *voluntary* opening of the boundary with others may – perhaps by enhancing a sense of social belonging -facilitate recovery.

Lee’s findings that bodily disturbances were less disturbing for Koreans than Americans point toward the possibility that a weakening of bodily boundaries may be more distressing for participants from egocentric cultures (54). Moreover, Markus and Kitayama’s work (1991) (36) on cultural construals of the self maintains that persons from sociocentric cultures experience the self differently and this has significant implications for how we construct emotion, cognition, and behavior. They suggest the self in collectivist cultures is interdependent with the surrounding context and defined through relationships with others (self in relation to others). Somasundaram (53) confirms that Sri Lankan Tamil culture is indeed sociocentric. Moreover, his work on the role of collective trauma in sociocentric cultures suggests that meaning attributed to experience of symptoms, beliefs about causation in psychopathology can differ by culture. In sociocentric contexts such as Sri Lanka, persons may be more concerned with the loss of social capital, cohesion, connectedness at the level of the community and these processes may be more relevant to post-traumatic recovery. Furthermore, Somasundaram suggests that social processes may influence pathophysiology to change bodily processes (52). Hence, it is possible that experiences and recovery processes of psychosis differ in sociocentric cultures. Hence, in the case of disrupted ipseity, it is possible that the interdependent or sociocentric self – which is arguably more open to the influence of others – may allow for greater connectedness and belonging, as this is culturally accepted and welcome. Further research is needed to establish how common such experiences of positive boundary weakening during ritual might be, explore whether similar experiences are found in western data and determine whether positive experiences of boundary weakening in psychosis is a universal or cultural phenomenon.

At the same time, participants also strengthened the boundaries of the self by emphasizing their impermeability, autonomy and differentiating themselves from others. In the moment of Siva’s ritual, we, for instance, can notice this co-presence of openness (letting himself be part of the ritual) and closedness (refusing to spit on the chicken). Thus, the boundaries of the self might be maintained by dynamically and intentionally shifting between experiencing the self both in relation to, and in opposition to, others. This is consistent with systemic, dialogical and phenomenological literature on schizophrenia which highlights that persons with schizophrenia need to strengthen the boundaries of the self by voluntarily affirming their separation, autonomy and differentiation from others (60–62) while also experiencing the need for connectedness, acceptance and belonging.

## Limitations

Many interviews collected for this study could not be used for IPA analysis as participants did not want to share psychotic experiences or did not provide detailed descriptions of their own experiences and preferred to talk about other persons, or other problems. Some participants did not describe boundary weakening. Participants were not used to research interviews, and their lack of trust may have influenced the content of their narrative. Finally, no women were included in this analysis. Female participants may have been afraid to disclose and describe symptoms of psychosis due to higher social stigma within a patriarchal society. Hence, threats to reputation may be more salient for women than for men.

## Conclusion

Pienkos (19) contends that disruptions of minimal selfhood in schizophrenia are not merely caused by psychological “processes within the individual”: basic processes of subjectivity are influenced by the external world.

Foremost, while previous research has understood boundary weakening and intersubjective processes associated with transitivism, i.e., the feeling of merging with others and losing one’s boundaries, as a consequence of the disruption of the minimal self and a loss of ipseity (13), this study suggests there may be a complex two-way relationship between disturbances of subjectivity and intersubjectivity. Although the loss of ipseity and hyper-reflexivity have been seen as consequences of the disruption of the minimal self, it is possible that disruptions of intersubjectivity such as invasiveness and hyperattunement precede, yield and shape the subjective ones. Whereas phenomenological concepts such as *intercorporeality*, *interaffectivity* (63), *openness* (64) and *open intersubjectivity* (65) corroborate this possibility on a theoretical level, our findings point to the need to further explore the relationship between intersubjective and subjective disruption in schizophrenia on the empirical level and understand how intersubjective experiences shape its emergence, experience and recovery in specific contexts.

Secondly, Hur and others (2014) (14) argue that there is a “glaring absence” of the role of culture as a contributor to self-awareness. This study suggests it is possible that there are cross-cultural differences in participants’ experiences during the disruption of the minimal self. Moreover, while self-awareness is probably universal, our study suggests that self-consciousness is structured, organized and narrated following cultural models of the self (35).

This study points to the need for culture specific models of self and psychosis. The notion of a sociocentric self is extremely broad and is perhaps Eurocentric, as the sociocentric self is defined in contrast to and negation of the ego-centric self. However, this theory enables us to explore differences in phenomenological experiences across multiple cultures and enrich our understanding of psychotic experiences and their recovery. In addition, local and culture-sensitive models of distress may better explain these participants’ close relationships during psychosis (66, 53, 67) and future studies should explore the phenomenology of psychosis through the lens of such models.

Finally, the study suggests that boundary weakening and recovery may be influenced by close relationships and that it is worth exploring the alteration of self-boundaries as they occur in the social context. Various authors (35, 68) suggest that the notion of autonomy as a therapeutic goal is a feature of the Western concept of the person. Moreover, psychopathology and difficulties with functioning in the Western context have been attributed to a failure to achieve full autonomy and define and achieve personal goals (35). Consistently with previous literature, our study suggests that the self can be seen as embedded in the socio-relational world (69); hence, the opening of boundaries and allowing the others to influence the participant may be experienced positively and facilitate connectedness and belonging. More research is needed to explore dialogical openness and closeness during experiences of boundary weakening and explore specific ways in which intersubjective experiences may facilitate the connectedness with others.

## Data availability statement

The original contributions presented in the study are included in the article/supplementary material, further inquiries can be directed to the corresponding authors.

## Ethics statement

Ethical review and approval was not required for the study on human participants in accordance with the local legislation and institutional requirements. The patients/participants provided their written informed consent to participate in this study. Written informed consent was obtained from the individual(s) for the publication of any potentially identifiable images or data included in this article.

## Author contributions

LG, EA, and LF: study conception and design. EA: data collection. EA, LG, and LF: analysis and interpretation of the findings. EA, LG, LF, and ST: draft manuscript preparation. All authors reviewed the results and approved the final version of the manuscript.

## Conflict of interest

The authors declare that the research was conducted in the absence of any commercial or financial relationships that could be construed as a potential conflict of interest.

## Publisher’s note

All claims expressed in this article are solely those of the authors and do not necessarily represent those of their affiliated organizations, or those of the publisher, the editors and the reviewers. Any product that may be evaluated in this article, or claim that may be made by its manufacturer, is not guaranteed or endorsed by the publisher.

## Footnotes

^1^The interviewer avoided using terms such as “psychosis” and “schizophrenia” during interviews as many patients were unfamiliar with medical language. In addition, the purpose of the study was to explore how patients experienced, described and made sense of their own symptoms, hence as researchers, we did not want to impose clinical language on participants. To elicit phenomenological descriptions of their psychotic experiences, the interviewer used general non stigmatising terms such as குழப்பம்்்் pronounced *kulappam* meaning turmoil, confusion. Another term they used was கஷ்டம் pronounced *kashtam* meaning trouble, difficulty.

^2^In the study of psychopathology, objective causal explanation soon reaches its limits (34). Conscious experience is the “research subject” of a phenomenological study and phenomenology describes how we perceive and make sense of the world, rather than what we perceive. Hence, in IPA, we explore how participants attribute causation to events rather than genetic or objective causation. Through double hermaneutics, we use causal language to capture our participants' meaning making of the relationship between external entities/others and their effect on self-experience.

^3^Here the participant appears to be describing what other people are thinking about him. According to others, the participant during the crisis is “like this,” i.e., he behaves in ways that are not socially acceptable (e.g., bringing stray animals home).
